# Effects of vasoactive drugs on crystalloid fluid kinetics in septic sheep

**DOI:** 10.1371/journal.pone.0172361

**Published:** 2017-02-23

**Authors:** Yuhong Li, Zheng Xiaozhu, Ru Guomei, Ding Qiannan, Robert G. Hahn

**Affiliations:** 1 Department of Anesthesiology, Shaoxing People's Hospital, Shaoxing, Zhejiang Province, PR of China; 2 Research Center, Shaoxing People's Hospital, Shaoxing, Zhejiang Province, PR of China; 3 The Department of Anesthesiology, Zhejiang Hospital, Hangzhou, Zhejiang Province, PR of China; 4 Research Unit, Södertälje Hospital, Södertälje, Sweden; Nanjing University Medical School Affiliated Nanjing Drum Tower Hospital, CHINA

## Abstract

**Purpose:**

Crystalloid fluid and vasoactive drugs are used in the early treatment of sepsis. The purpose of the present study was to examine how these drugs alter plasma volume expansion, peripheral edema, and urinary excretion.

**Methods:**

Twenty-five anesthetized sheep were made septic by cecal puncture and a short infusion of lipopolysaccharide. After 50 min, a slow infusion of isotonic saline was initiated: the saline either contained no drug, norepinephrine (1 μg/kg/min), phenylephrine (3 μg/kg/min), dopamine (50 μg/kg/min), or esmolol (50 μg/kg/min). Ten min later, 20 mL/kg Ringer´s lactate solution was given over 30 min. Central hemodynamics, acid-base balance, and the urinary excretion were monitored. Frequent measurements of the blood hemoglobin concentration were used as input in a kinetic analysis, using a mixed effects modeling software.

**Results:**

The fluid kinetic analysis showed slow distribution and elimination of Ringer´s lactate, although phenylephrine and dopamine accelerated the distribution. Once distributed, the fluid remained in the peripheral tissues and did not equilibrate adequately with the plasma. Overall, stimulation of adrenergic alpha_1_-receptors accelerated, while beta_1_-receptors retarded, the distribution and elimination of fluid. A pharmacodynamic E_max_ model showed that Ringer´s lactate increased stroke volume by 13 ml/beat. Alpha_1_-receptors, but not beta_1_-receptors, further increased stroke volume, while both raised the mean arterial pressure. Modulation of the beta_1_-receptors limited the acidosis.

**Conclusions:**

Stimulation of adrenergic alpha_1_-receptors with vasoactive drugs accelerated, while beta_1_-receptors retarded, the distribution and elimination of fluid. The tendency for peripheral accumulation of fluid was pronounced, in particular when phenylephrine was given.

## Introduction

Crystalloid fluid loading and the administration of vasoactive drugs are essential steps in the early treatment of septic patients with suspected hypovolemia and tissue hypoperfusion [1, 2]. The treatment for an adult consists of an infusion of at least 30 ml/kg of crystalloid fluid such as Ringer´s lactate. If the hypovolemia and hypotension is not resolved by volume loading, the treatment should be augmented by administration of norepinephrine, phenylephrine, or dopamine [3, 4].

In healthy sheep, vasoactive drugs markedly change the distribution and elimination of crystalloid fluid, thereby altering the plasma volume expansion, urinary excretion, and the risk of peripheral edema [5]. However, the interactions between fluid and vasoactive drugs have not been studied with respect to sepsis. This type of study is of potential importance in the search for optimal combinations of crystalloids and vasoactive agents.

The purpose of the present study was to use volume kinetics to describe the distribution and elimination of crystalloid fluid in an experimental sepsis model, based on serial measurements of the hemodilution during and after an infusion [6–8], and how this is influenced by various vasoactive drugs (norepinephrine, phenylephrine, dopamine, and esmolol). Volume kinetics is pharmacokinetics for infusion fluids and has been applied in approximately 50 studies of various fluid therapies [6]. Not until recently has the population kinetic (mixed models) approach been developed that is necessary for the analysis of the relationship between fluid distribution and adrenergic stimulation [8].

## Materials and methods

### Animals

Twenty-five healthy male sheep weighing 14–26 kg (mean, 20 kg) were studied. The protocol and the experimental procedures were approved by the Animal Care and Use Committee of the Shaoxing People’s Hospital (PR of China, approval number ZJU20140252), and the study was conducted in adherence with the Guide for Care and Use of Laboratory Animals.

### Anesthesia and surgical preparation

After an overnight fast, anesthesia was induced with propofol (5 mg/kg). The animals were intubated via tracheotomy and ventilated at a tidal volume of 10 ml/kg and respiratory rate of 15 breaths per minute. The anesthesia was maintained with 1.5–2.5% sevoflurane and intermittent doses of sufentanil (0.6 μg/kg) and cisatricurium (0.2 mg/kg), as needed.

In a sterile operating environment, a 3-lumen vascular catheter was placed in the right internal jugular for drug administration and fluid infusion, as well as for registration of the central venous pressure (CVP). One femoral artery was cannulated for measurement of blood pressure and for sampling of blood. A middle laparotomy was performed and a cystostomy catheter was placed in the bladder for collection of urine.

### Sepsis model and vasoactive drugs

Sepsis was created by cecal ligation and puncture, combined with a 10-min intravenous (i.v.) infusion of 0.5 mg/kg of lipopolysaccharide (LPS). Fifty minutes after the infusion of LPS, a continuous infusion was initiated consisting of 10 ml/kg 0.9% sodium chloride containing no vasoactive drug (control), dopamine (50 μg/kg/min), noradrenaline (1 μg/kg/min), phenylephrine (3 μg/kg/min), or esmolol (50 μg/kg/min). The rates of administration of phenylephrine and dopamine were chosen to agree with a previous study of fluid kinetics in sheep [5]. The dose of norepinephrine corresponds to the mean dose used in clinical trials of septic shock. Esmolol is a beta_1_-receptor blocker and is not used to support hemodynamics in septic patients. This drug was included to increase the width of our manipulation of the beta_1_-receptors and for its ability to increase urinary excretion, and the dose corresponds to the one used in a study of volume kinetics during laparoscopic surgery [7].

Ten minutes after initiation of the infusion of a vasoactive drug, plasma volume expansion was induced by infusing 20 ml/kg of Ringer’s lactate (Pharmacia-Baxter, Shanghai, China) over a 30 min period. The vasoactive drugs were maintained for 150 min, and then the experiment was ended and the animals were sacrificed.

**Fig 1:**
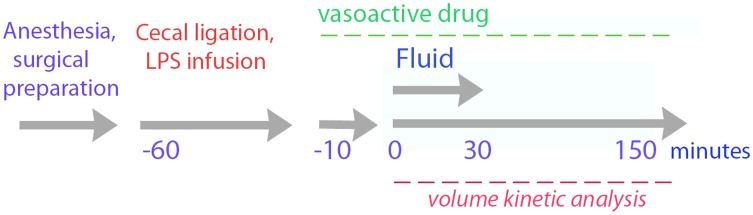
Schematic drawing of the time-line in the study.

The phases of the study protocol are illustrated in Fig 1.

### Measurements

The arterial cannula was connected to a FloTracTM sensor and the sensor data were sent to a Vigileo monitor (Software version 3.6; Edwards Lifesciences, Irvine, CA) for measurements of cardiac output (CO) and stroke volume (SV). These data were calibrated against the cardiac index yielded by thermodilution in anesthetized sheep [9].

Monitoring also included mean arterial pressure (MAP), central venous pressure (CVP), electrocardiography, and heart rate. All results were displayed on the multifunction monitor (Datex-Ohmeda, Hoevelaken, The Netherlands) and saved digitally. Arterial blood samples (2 ml each) were collected every 5 min for 60 min after the starting the infusion of LPS and again during the first 60 min after infusion of Ringer’s solution, and then every 10 min during the following 90 min.

The total blood hemoglobin (Hb) concentration was measured on a GEM Premier 3000 instrument (Instrumentation Laboratory, Lexington, IL). Duplicate samples collected at baseline ensured a coefficient of 1.5%. Serum lactate and base excess (BE) were also measured at the same time. Urine outputs were measured 60 min after the cecal puncture and at the end of the study.

All animals were sacrificed by an injection of potassium chloride upon completion of the experiment.

### Kinetic analysis

Hemodilution is the inverse of the blood water concentration [10] and was used as the input function in the kinetic analysis because Ringer´s lactate is 99% water. The plasma dilution was then fitted to two-volume kinetic model with micro-constants, and the influence of covariates (adrenergic receptor stimulation or blockade) was tested [7].

**Fig 2:**
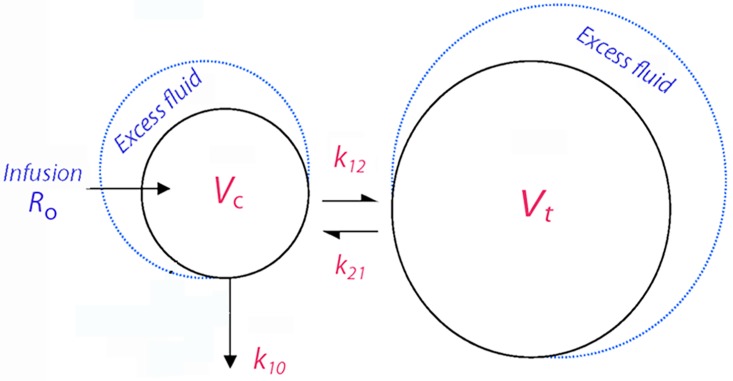
The fluid kinetic model used to analyze the dilution of arterial plasma. ***Abbreviations*:** *V*_c_ and *V*_t_ = central and peripheral fluid space expanded by infused fluid to *v*_c_ and *v*_t_. *k*_12_ and *k*_21_ = rate constants governing the fluid transfer from *v*_c_ and *v*_t_ and vice versa. *k*_10_ = elimination rate constant.

Fluid was infused at rate *R*_o_ to expand the volume of the central body fluid space (i.e. the plasma volume) from *V*_c_ to *v*_c_. The rate of elimination was given as the product of the volume expansion of *V*_c_ and an elimination rate constant, *k*_10_. The distribution to the peripheral body fluid space *V*_t_ (i.e. the interstitial fluid space) was governed by a rate constant *k*_12_ and its return from *v*_t_ to *v*_c_ by another rate constant, *k*_21_ (Fig 2).

The differential equations are:

$$\begin{array}{l} dv_{\text{c}}/dt=R_{\text{o}}-k_{10}(v_{c}-V_{c})-k_{\text{b}}(v_{c}-V_{c})-k_{12}(v_{c}-V_{c})+k_{21}(v_{\text{t}}-V_{\text{t}})\\ dv_{t}/dt=k_{12}(v_{c}-V_{c})-k_{21}(v_{\text{t}}-V_{\text{t}}) \end{array}$$

The Hb-derived fractional plasma dilution was used to indicate the volume expansion of *V*_c_ resulting from the infusion. This provides a linear relationship between the added fluid volume and the change in Hb in an expandable fluid space [6]. Hence:

$$(v_{\text{c}}-V_{\text{c}})/V_{\text{c}}=((Hb/hb)-1)/\text{​}(1-\text{hematocrit)}$$

The symbols in capital letters denote baseline values. The "false" plasma dilution caused by the blood sampling was corrected by a mathematical procedure explained elsewhere [6]. On assuming that Hb is evenly distributed in the circulation, the Hb-derived fractional plasma dilution also indicates the expansion of the plasma volume.

The primary parameters in the model (*V*_c_, *k*_12_, *k*_21_, and *k*_10_) and their covariates were estimated using the Phoenix software for nonlinear mixed effects (NLME), version 1.3 (Pharsight, St. Louis, MO).

The analysis was performed in four parts. First, the four kinetic parameters were estimated separately for each of the study groups separately. The distribution of the infused fluid between body compartments was then simulated based on the best estimates of these parameters, using Matlab R2012b (Math Works Inc., Natick, MA).

Second, all experiments were pooled, and *base model* was developed which included the four kinetic parameters in the model.

Third, covariates in the form of various degrees of receptor stimulation were then added in sequence to the base model, which eventually formed the *final model* [8–11]. Receptor effects were included the final model if they were statistically significant (95 confidence interval, CI, did not include 1.0) and their coefficient of variation was < 30%.

The degree of adrenergic receptor effect assumed to be produced by the various drugs is shown in Table 1. The strength of all statistically significant receptor effects (being either blockade -1, no effect 0, slight stimulation 1, stronger stimulation 2, or strongest stimulation 3) were applied simultaneously in the final analysis of the full model. To stabilize these calculations, the measured urinary excretion in each experiment was set equal to *k*_10_ (the elimination) in that experiment.

**Table 1:** The level of stimulation of receptors used in the volume kinetic analysis. Zero means no effect, +3 strong effect, and -1 means inhibition.

Drug treatment	Alpha_1_-adrenergic receptors	Beta_1_-adrenergic receptors	Dopaminergic receptors
None (control)	0	0	0
Norepinephrine	3	2	0
Phenylephrine	3	0	0
Dopamine	1	1	1
Esmolol	0	–1	0

Based on Reference [15].

Fourth, the effect of plasma dilution on key hemodynamic parameters was fitted to a pharmacodynamic linear effect model (E_max_ model) having the following appearance:

$$E=E_{\text{o}}\text{ + }\frac{E_{\text{max}}((v_{\text{c}}\text{-}V_{\text{c}}\text{)/}V_{\text{c}}\text{)}}{EC_{50}((v_{\text{c}}\text{-}V_{\text{c}}\text{)/}V_{\text{c}}\text{)}}$$

where *E* is effect, *E*_o_ is the effect at baseline, *E*_max_ is the maximum effect, and *EC*_50_ is the fractional plasma dilution required to cause 50% of the full effect.

### Statistics

Demographic and hemodynamic data were reported as the mean (standard deviation, SD) and the kinetic data as the mean (95% confidence interval, CI). Changes in parameters were compared by the paired *t* test or the Wilcoxon matched-pair test. The effect of adrenergic receptor stimulation on the hemodynamics was evaluated by one- and two-way ANOVA. Correlations were studied by linear regression analysis, where r = correlation coefficient. *P* < 0.05 was considered statistically significant.

## Results

Our evaluation describes the overall effects of sepsis and fluid of the hemodynamics, analyses the fluid kinetics for each of the five subgroups separately as well as the fluid kinetics for all 25 animals together, in which the latter used the levels of adrenergic stimulation as covariates. Finally, selected analyses of the independent effects of fluid treatment and adrenergic stimulation on hemodynamics and acid-base balance were made.

### Hemodynamics

**Fig 3:**
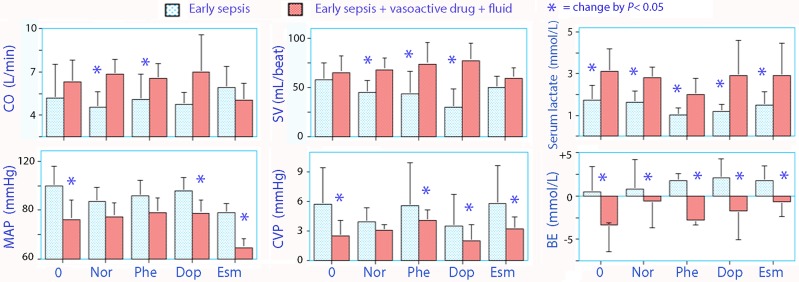
Hemodynamics. Early sepsis (-60 to -10 min) compared with treatment using vasoactive drugs and fluid (0 to 150 min). Data are mean (SD) based on patient mean values during the indicated period of time. Due to the small number in each group (n = 5), statistical comparions were made by using the Wilcoxon matched-pair test. * indicates statistical significance. ***Abbreviations*:** CO = cardiac output, SV = stroke volume, MAP = mean arterial pressure; CVP = central venous pressure, BE = base excess; Nor = norepinephrine, Phe = phenylephrine, Dop = dopamine, Esm = esmolol.

During the 50 min of early sepsis alone, MAP gradually decreased from 97 (17) to 64 (20) mmHg, while CO rose from 4.0 (1.8) to 5.7 (2.6) L/min (paired *t* test, both *P*< 0.001). SV increased only slightly. The plasma was concentrated by 9.5% (median; 95% CI 4.4–12.7) (Fig 3).

Compared with early sepsis alone, early sepsis plus adrenergic drug and fluid treatment was associated with a higher mean CO, SV, and serum lactate (*P*< 0.001), whereas the MAP, CVP, and base excess decreased (*P*< 0.001, Table 2).

**Table 2:** Hemodynamics and biochemical evidence of acidosis. Early sepsis (-60 to -10 min) is compared to the time period when fluid and vasoactive drug were given (0 to 150 min). These data are plotted in Fig 3. Each group contained 5 animals.

	Control	Nor	Phe	Dop	Esm
**CO (L/min)**					
**Early sepsis**	5.2 (2.4)	4.6 1.1)	5.0 (1.7)	4.8 (0.9)	5.9 (1.5)
**Drug + fluid**	6.3 (1.5)	6.9 (1.0)	6.6 (1.2)	7.0 (2.6)	5.0 (1.2)
**SV (mL/beat)**					
**Early sepsis**	58 (16)	46 (6)	44 (15)	31 (13)	55 (12
**Drug + fluid**	66 (17)	68 (12)	74 (22)	77 (18)	60 (11)
**Serum lactate (mmol/L)**					
**Early sepsis**	1.7 (0.7	1.6 (0.6)	1.0 (0.3)	1.2 (0.3)	1.5 (0.6
**Drug + fluid**	3.1 (1.1)	2.8 (0.4)	2.0 (0.8)	2.9 (1.7)	2.9 (1.6)
**MAP (mmHg)**		)			
**Early sepsis**	100 (10)	87 (6)	92 (6)	96 (6)	78 (4)
**Drug + fluid**	72 (16)	75 (12	78 (12)	77 (11)	49 (7)
**CVP (mmHg)**					
**Early sepsis**	5.8 (3.7)	3.9 (1.4)	5.6 (4.4)	3.6 (3.2)	5.8 (3.8)
**Drug + fluid**	2.5 (1.6)	3.1 (0.6)	4.1 (1.0)	5.8 (3.8)	3.2 (1.2)
**BE (mmol/L)**					
**Early sepsis**	0.5 (2.9)	0.8 (3.4)	1.8 (0.8)	2.1 (2.2)	1.8 (1.7)
**Drug + fluid**	–3.3 (3.1)	–0.6 (3.1)	–2.8 (0.5)	–1.7 (3.3)	–0.7 (1.6)

***Abbreviations*:** CO = cardiac output, SV = stroke volume, MAP = mean arterial pressure; CVP = central venous pressure, BE = base excess; Nor = norepinephrine, Phe = phenylephrine, Dop = dopamine, Esm = esmolol.

### Kinetics in the five groups

**Fig 4:**
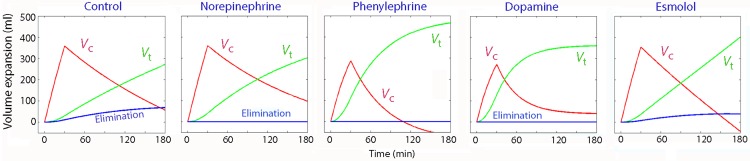
Fluid distribution. Volume expansion of the central fluid space (*V*_c_, the plasma), the peripheral fluid space (*V*_t_) and the excreted urine (the elimination) during infusion experiments with 20 mL/kg of Ringer´s lactate over 30 min in septic sheep who were also given a vasoactive substance. Computer simulation using the parameter estimates shown in Table 3.

A separate population kinetic analysis was first made separately for the controls and for each of the 4 groups of 5 sheep that received a specific vasoactive drug. The distribution rate constant (*k*_12_) was high for in the phenylephrine and dopamine, but low in the other groups. The kinetic constant governing the rate of for re-distribution (*k*_21_) was negative in most groups (Table 3). which resulted in marked peripheral accumulation of infused fluid (Fig 4).

**Table 3:** The patterns of receptor stimulation used in the volume kinetic analysis.

	Control	Norepinephrine	Phenylephrine	Dopamine	Esmolol
*V*_c_ (L)	1.32 (0.97–1.68)	2.65 (1.96–3.35)	1.34 (1.25–1.44)	1.60 (1.18–2.03)	1.80 (1.36–2.24)
*k*_*1*2_ (10^−3^ min^-1^)	5.2 (0.3–10.0)	7.2 (3.3–11.1)	22.3 (17.0–27.7)	28.5 (22.3–34.6)	6.3 (2.4–10.1)
*k*_21_ (10^−3^ min^-1^)	-3.9 (-9.5 to 1.8)	-1.2 (-1.9 to -0.6)	-4.1 (-5.0 to -3.1)	3.0 (1.9–4.1)	-6.8 (-11.7 to -2.0)
*k*_10_ (10^−3^ min^-1^)	2.0 (0.5–3.5)	< 0.01	< 0.01	< 0.01	1.4 (0.5–2.4)

The best estimate of each parameter is shown together with its 95% confidence interval. The additive within-subject residual error structure and the extended least squares (ELS) analysis model without consideration of the measured urinary excretion yielded the most precise estimates of the four parameters.

### Covariate analysis

A population kinetic analysis was then performed based on all infusion experiments. The covariance analysis was most successful with a linear model, where the strength of each receptor effect, according to Table 1, was applied as a continuous variable. There were five covariate effects: *k*_12_ and *k*_10_ were increased by stimulation of the alpha_1_-receptors but decreased by the beta_1_-receptors. In addition, the beta_1_-receptors increased *V*_c_. No significant covariate effect was found for the dopamine receptors.

The complete set of parameter estimates are given in Table 4. The best estimates in the model were then modified with regard to *V*_c_, *k*_12_, and *k*_10_, as follows:

$$\begin{array}{l} V_{\text{c}}=1.70\;(1+0.12{\text{ beta}}_{1})\\ k_{12}=6.4\text{ x }10^{-3}\;(1+1.45{\text{ alpha}}_{1})\;(1-0.33{\text{ beta}}_{1})\\ k_{10}=0.5\text{ x }10^{-3}(1+1.93{\text{ alpha}}_{1})\;(1-0.32{\text{ beta}}_{1}) \end{array}$$

where the digits applied for assumed strength, according to Table 1, are used to replace alpha_1_ and beta_1_ in the equations.

**Table 4:** Pharmacokinetic and pharmacodynamic parameters in the final model.

	Covariate	Best estimate (95% CI)
**Kinetic parameter**		
tv*V*_c_ (L)		1.70 (1.47–1.93)
tv*k*_12_ (10^−3^ min^-1^)		6.4 (3.8–9.0)
tv*k*_21_ (10^−3^ min^-1^)		-4.1 (-6.0 to -2.1)
tv*k*_10_ (10^−3^ min^-1^)		0.50 (0.24–0.76)
**Covariate effect**		
tv*k*_12_	Alpha_1_ stimulation	0.45 (0.24–0.65)
tv*k*_10_	Alpha_1_ stimulation	1.93 (1.07–2.80)
tv*V*_c_	Beta_1_ stimulation	0.12 (0.05–0.19)
tv*k*_12_	Beta_1_ stimulation	-0.32 (-0.49 to –0.14)
tv*k*_10_	Beta_1_ stimulation	-0.33 (-0.51 to –0.14)
**E**_**max**_**, ΔSV**		
tvE_0_		13 (6–20)
tvE_50_		9 (4–14)
tvE_max_		49 (-6 to 106)

tv = typical value, CI = confidence interval,

ΔSV = the change in stroke volume since the onset of fluid infusion.

**Fig 5:**
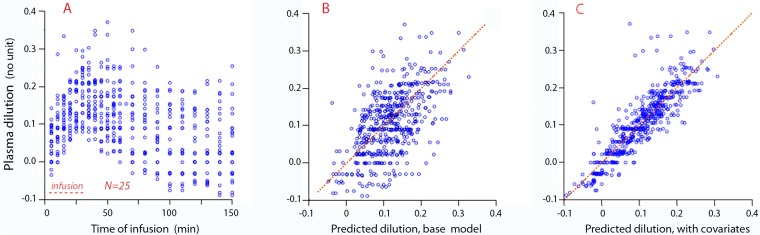
The curve-fitting procedure. **A:** The measured plasma dilution over time in all sheep, regardless of vasoactive drug. **B:** The plasma dilution predicted from the base model (four parameters only) without consideration of covariates (drug effects), and **C:** The plasma dilution predicted from the final model with covariate effects.

The measured plasma dilution when all data were pooled is shown in Fig 5A. The agreement between the measured and predicted plasma dilution according to the base model is given in Fig 5B and the same comparison, but with consideration taken of the covariates, is made in Fig 5C.

**Fig 6:**
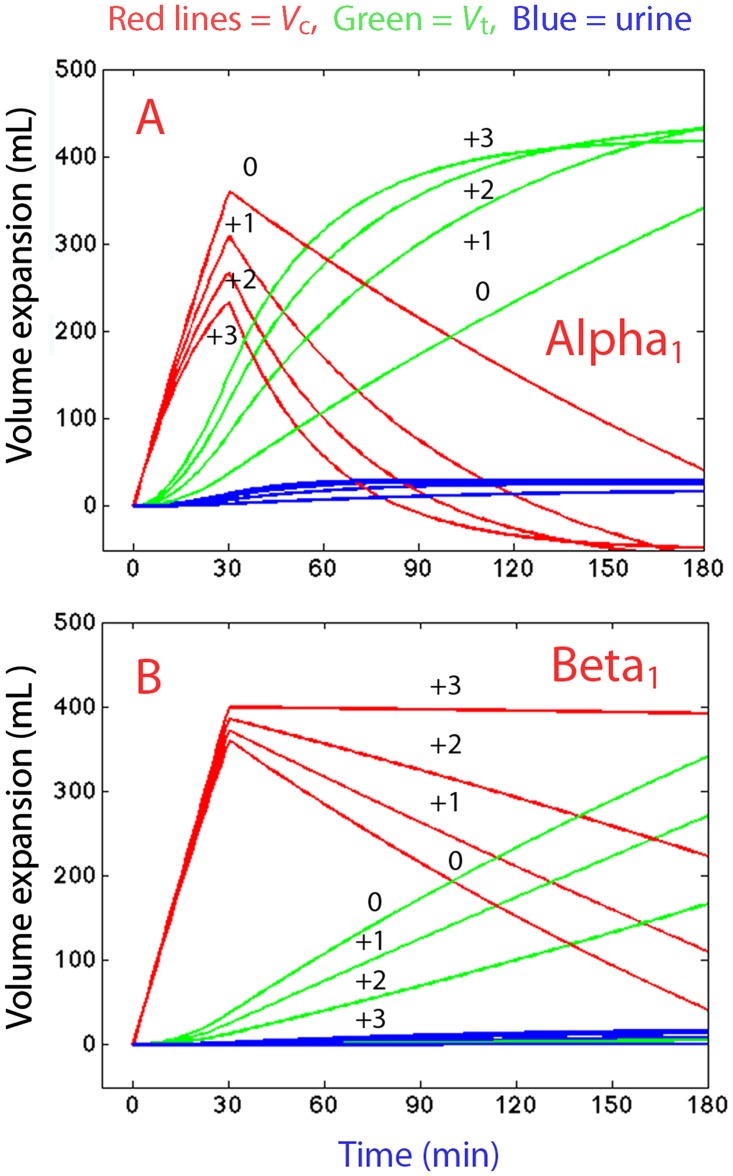
Simulation of adrenergic effects on fluid distribution. Distribution of 20 ml/kg Ringer´s lactate infused over 30 min in sheep weighing 20 kg, depending on the degree of alpha_1_-adrenergic **(A)** and beta_1_-adrenergic **(B)** stimulation, graded between 0 and +3, according to Table 1. The kinetic parameters shown in Table 3 were used for the simulation.

Fig 6 illustrates how changes in the degree of adrenergic stimulation would alter the distribution of crystalloid fluid according to the analysis made. *V*_c_ would become less expanded, while *V*_t_ would be more expanded, the stronger the stimulation of the alpha_1_-adrenergic receptors (Fig 6A). The effect would be the opposite for stimulation of the beta_1_-adrenergic receptors (Fig 6B).

### Urinary excretion

The urinary excretion (the elimination) was small in all groups (Figs 4 and 6). During early sepsis alone, the median urine flow was 0.20 ml/min (95% CI, 0.16–0.40). During the treatments with a vasoactive drug and fluid, the values were quite similar, at 0.16 ml/min (0.13–0.31).

### Measures of effect

The E_max_ model analysis showed that the fluid treatment as such raised SV by 13 ml/beat, but further increases in response to increasing plasma dilution were slight (Table 3, bottom). Similar findings were observed for MAP (data not shown).

Two-way ANOVA was used to examine whether the adrenergic stimulation affected the hemodynamic parameters in addition to fluid alone. Alpha_1_-receptors raised SV by ≈15 ml/beat (*P*< 0.001) whereas the beta_1_-receptors did not significantly affect SV. Stimulation of these receptors raised MAP by ≈15 mmHg (*P*< 0.0001 and *P*< 0.02, respectively) without an additive effect; this was 2–3 more than with fluid alone. A greater hemodynamic effect for stronger receptor stimulation potency, as graded according to Table 1, could not be discerned.

Modulation of the beta_1_-receptors, but not of the alpha_1_-adrenergic, prevented a reduction of BE [–0.7 (2.7) mmol/L *versus* –2.9 (2.4) for the others, *P*< 0.0001] and of pH [7.38 (0.08) *versus* 7.33 (0.08); *P*< 0.001)]. In contrast, dopamine receptors offered marginal prevention [–1.2 (3.0) *versus* –1.7 (2.8) mmol/L, *P* = 0.08).

## Discussion

The animal model used here created a moderately severe sepsis with hyperkinetic circulation and was used to investigate the effect of vasoactive drugs on the kinetics of Ringer´s lactate. The infused fluid expanded a central fluid space (the plasma) of between 1.3 and 2.7 L, which is large when considering the small size of these animals (20 kg). The distribution of fluid from the central to the peripheral fluid space in septic sheep without vasoactive drug was quite slow, when compared to previous work in conscious healthy sheep [9]. A more normal rate of distribution (*k*_12_ 30–50 10^−3^ min^-1^) was achieved with phenylephrine and dopamine.

A slow distribution could be regarded as a benefit, as it increases the plasma volume expansion resulting from the infusion. Cardiac preload is then better maintained due to the larger fraction of the infused fluid that remains in the circulating blood. Moreover, oxygenation of peripheral tissues will be better preserved because the diffusion distance becomes smaller when the edema in peripheral tissues (*V*_t_) is less pronounced [12]. However, the overall impact of the fluid infusions on edema must be interpreted with a knowledge of both *k*_12_ and *k*_21_, where normal fluid exchange implies that the former rate constant should be about twice as high as the latter [7, 8]. However, in this study the fluid exchange between *V*_c_ and *V*_t_ was clearly not normal. The rate constant *k*_21_ even attained negative values, which has previously only been described for the "transurethral resection syndrome" [13].

The negative value denotes that fluid accumulated in peripheral tissues without equilibrating adequately with the plasma, indicative of a shock-like situation. The combination of a normal *k*_12_, negative *k*_12_, and low *k*_10_ strongly promotes the development of peripheral edema and, in our simulations, even late hypovolemia (Fig 4). Edema was a problem encountered with all the vasoactive drugs, but particularly with phenylephrine. However, most of the slow elimination (low *k*_10_) can be understood on the basis of the use of general anesthesia [14], and the hypovolemia caused by capillary leakage during the early phase of the sepsis, which amounted to 10% of the plasma volume.

Better insight into the fluid kinetics was sought by performing the full population kinetic analysis where the relative effects of the drugs on adrenergic receptors were used as covariates (Table 1). Population kinetic analysis has gradually become a scientific and industrial standard procedure in drug development [11], and it can be applied to fluid volume kinetics as well [8]. Using this approach, the distribution and elimination of Ringer´s lactate in all experiments could be analyzed with higher precision than was possible with a separate analysis for each drug, and even in a single run. An analysis of the individual contribution of different adrenergic receptors on fluid kinetics could also be made.

The results show that alpha_1_-adrenergic receptors accelerated, while beta_1_-adrenergic receptor stimulation retarded, the distribution of crystalloid fluid. The magnitude of these influences could be quantified (Table 3) and illustrated graphically (Fig 6). The pronounced edema found with phenylephrine can then be understood from the strong alpha_1_-receptor effect, but lack of beta_1_-stimulation, associated with the use of this drug. Moreover, the acidosis was less pronounced when the beta_1_-receptors were stimulated, which confirms that maintaining plasma volume expansion (*V*_c_) at the expense of extravascular fluid accumulation (*V*_t_) is beneficial to oxygenation of the tissues [12].

The present findings also indicate that alpha_1_-receptor stimulation increased while beta_1_-adrenergic receptor stimulation decreased the urinary excretion. Hence, these effects were opposite to those exerted by the adrenergic receptors on the rate of fluid distribution. They are supported by a previous study in conscious sheep [5], and also by a study in anesthetized women undergoing laparoscopic gynecological surgery [7]. In the present study the increase in urinary excretion caused by modulation of the adrenergic receptors, was statistically significant but still quite small, which is probably due to the overall inhibition of *k*_10_ caused by a combination of sepsis, general anesthesia, and hypovolemia. Our previous work with non-septic sheep [5], and humans [7], suggests that the influence of adrenergic receptors on the urinary excretion would take priority over their effect on fluid distribution, if the strong context-sensitive inhibition of diuresis had been less pronounced.

The pharmacodynamic analyses suggest that the effects of volume expansion (plasma dilution) on the changes in SV and MAP were quite small. The adrenergic receptors had a relatively stronger influence on the hemodynamics. Stimulation of alpha_1_- and/or beta_1_-adrenergic receptors increased MAP, but only the alpha_1_-adrenergic receptor stimulation increased SV. Interestingly, modulation of the beta_1_-adrenergic receptors limited the acidosis, which is an effect that should be evaluated further.

Simulations of fluid distribution, as depicted in Fig 3, requires only the three rate constants (*k*_12_, *k*_21_ and *k*_10_), while *V*_c_ can be regarded as a conversion factor between plasma dilution and plasma volume. The size of *V*_c_ represents the plasma volume but, more precisely, the body fluid volume that equilibrates very quickly with the plasma water in arterial blood. Plots created using these three rate constants illustrate how the body handles the fluid, with a barrier (probably the interstitial fluid gel) that retards distribution between *V*_c_ and a peripheral space, *V*_t_ [8]. The size *V*_t_ is not given directly from the model, but it can be calculated in retrospect as *k*_12_ *V*_c_ / *k*_21_. Given this equation, we can understand the degree of difficulty in interpreting a negative rate constant. The size of *V*_t_ then attains a negative value, which shows that fluid is virtually suctioned into *V*_t_, without having clearly defined borders.

From a translational perspective, the results of the present study suggest that crystalloid fluid given in an early phase of sepsis has a marked tendency to accumulate in extravascular, peripheral tissues. Drugs that exert a strong stimulating effect on beta_1_-adrenergic receptors help to limit this aberrant fluid distribution, and also reduce the systemic acidosis that is typical of sepsis. The beneficial influence of beta_1_-adrenergic receptor stimulation on the distribution of infused fluid corroborates earlier findings in conscious sheep [5]. Alpha_1_- and beta_1_-adrenergic receptor stimulation both acted to sligthly raise MAP, but a strong and preferential beta_1_-adrenergic effect could be considered to be a more beneficial characteristic of any vasoactive drug used during sepsis.

Our study has several limitations. The current international consensus guidelines for sepsis state that vasopressors therapy should be initiated as an adjunct therapy when hypovolemia and hypotension is not resolved through volume resuscitation. Here, the infusion of vasopressor was initiated 10 min before volume loading, as the purpose was to examine how the vasopressors affect fluid kinetics. The grading of the strength of the drugs on adrenergic receptors was based on literature that relates to non-septic, conscious subjects [15]. This grading, as given in Table 1, is subjective but still widely accepted in medicine. The computer simulations in Fig 6 may still seem somewhat speculative, although they should still illustrate trends in how adrenergic receptors alter fluid kinetics in sepsis. Only five animals were studied in each group, which limited the possibility of comparing the groups using statistics (Table 2), but the power of the kinetic analysis was markedly greater when comprising all animals (Table 3). The experiments were performed under general anesthesia, which alters the hemodynamics so that the responses to volume and pressor agents may differ compared with the responses in the conscious or sedated state. The FloTrac/Vigoleo is not marketed for use in sheep, although it is used for educational purposes in this animal. Therefore, we calibrated the data on the cardiac index at baseline in order to agree with the cardiac index obtained by thermodilution in a previous study [9].

Only one dose of each drug was tested. The results still provided information about how fluid kinetics varies with the degree of the alpha- and beta-receptor stimulation, as these characteristics differed greatly between the drugs used. The administered amounts of drug are not as high as they might seem, because extrapolation of doses between species should be made by considering the "effective body size," which is the 2/3 power of the body weight [5]. However, the capacity of esmolol to increase the urinary excretion proved to be of limited value due to the strong overall inhibition of diuresis in this setting.

In conclusion, infusion of vasoactive drugs in septic sheep with moderate hypovolemia and hypotension showed that stimulation of alpha_1_-receptors accelerated, while beta_1_-receptors the retarded, the distribution and elimination of Ringer´s lactate. Peripheral edema was pronounced due to the virtual absence of redistribution of fluid from peripheral tissues. The effectiveness of plasma volume expansion for improving hemodynamics was limited. Modulation of beta_1_-receptors reduced acidosis.

## Supporting information

S1 Dataset The excel file with all original data. (XLS)
